# Suicidal Behavior and Its Relationship with Postmortem Forensic Toxicological Findings

**DOI:** 10.3390/toxics10060319

**Published:** 2022-06-11

**Authors:** Aurelia Collados-Ros, Carmen Torres-Sánchez, María Dolores Pérez-Cárceles, Aurelio Luna, Isabel Legaz

**Affiliations:** 1 Department of Legal and Forensic Medicine, Biomedical Research Institute (IMIB), Regional Campus of International Excellence “Campus Mare Nostrum”, Faculty of Medicine, University of Murcia, 30100 Murcia, Spain; aurelia.c.r@um.es (A.C.-R.); mctorres@um.es (C.T.-S.); mdperez@um.es (M.D.P.-C.); aurluna@um.es (A.L.); 2 Pathology Service, Institute of Legal Medicine and Forensic Sciences, 30100 Murcia, Spain

**Keywords:** alcohol, autopsies, drugs of abuse, forensic toxicology, psychotropic drugs, suicides

## Abstract

Suicide affects all sociodemographic levels, age groups, and populations worldwide. The factors that can increase the risk of suicidal tendencies are widely studied. The aim of this study was to analyze the types and combinations of toxics found in fatal suicide victims with different suicide mechanisms. A total of 355 autopsies were retrospectively studied, and 26 toxics were determined and related to mechanisms of suicide. Hanging (55%), drug overdose (22.7%), and jumping from a height (17.8%) were most represented suicide mechanisms with positive toxicology. Hanging was the most represented in men (50.3%; *p* = 0.019), while jumping from a height was more represented in women (29.7%, *p* = 0.028). Drugs of abuse were the most frequent toxics found in men (55.5%; *p* < 0.001), while medicines were the most frequent type found in women (70.3%, *p* < 0.001). Alcohol, nordiazepam, cocaine, and venlafaxine were the most consumed toxics. Benzodiazepines and venlafaxine were found in suicides involving drug overdose, hanging, and jumping from a height. In conclusion, most suicides were associated with drug abuse in men. Hanging was more represented in men and jumping from a height in women. Alcohol was present in combination with other toxics and medicines. The toxicological analysis is fundamental to understanding consumption patterns and establishing strategies and protocols for detecting and preventing suicide.

## 1. Introduction

Suicide is one of the leading causes of death worldwide, making it a serious medical and social problem [1]. Suicide affects all sociodemographic levels, age groups, and populations [2,3,4]. Great effort is currently being made to establish the factors contributing to or increasing the risk of suicide [5,6,7]. Different studies confirm that suicide attempts are generally associated with mental illness, including affective or psychiatric disorders, depression, hopelessness, impulsivity, and alcohol and/or substance abuse [8,9,10]. However, alcohol and substance abuse are strongly associated with suicidal ideation but only weakly predict suicide attempts among ideators [11,12,13,14,15,16,17]. The associations between psychotropic medications and the risk of suicide are a recurring topic of discussion [18,19]. Antipsychotic agents appear to have protective effects in both non-fatal suicides and complete suicidal acts, but the possible anti-suicide or pro-suicide properties of antidepressants remain inconclusive [20,21,22,23]. Nevertheless, it was observed that people treated with medications were 9.6 times more likely to attempt suicide during the hours they were intoxicated [24]. Suicidal mixing of drugs such as opioids or barbiturates increases the risk that a suicide attempt will eventually be consummated [25].

Despite the advances in understanding suicidal behavior, there are still many biological responses to resolve [26,27,28]. The role of signal transduction and molecular systems in medicated patients with major depression or drug abuse and/or suicidal behavior is being investigated [29,30]. Given the sex differences in suicide rates, some studies hypothesize that the pattern of brain gene expression is different in male and female suicides, and changes in the expression of sex-specific genes have been identified in the prefrontal cortex of suicide bombers [31,32,33]. Carrying out toxicological analysis in cases of fatal suicide is a crucial piece to understanding consumption patterns and establishing strategies and protocols for the detection and prevention of suicide.

The aim of this study was to analyze the types and combinations of toxics found in fatal suicide victims with different suicide mechanisms to increase knowledge and establish policies and protocols for detecting, preventing, and avoiding suicide behavior.

## 2. Materials and Methods

### 2.1. Suicide Enrolment, Data Acquisition, and Mechanisms of Suicide

**Figure 1:**
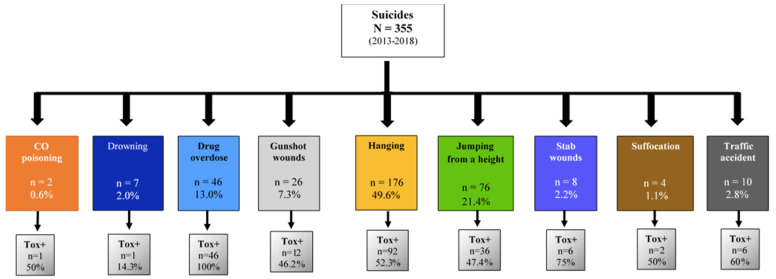
Scheme of the total suicides revised in this study classified by the suicide mechanism and the presence of toxic substances in the cadaver. CO, carbon monoxide; N, total sample size; n, sample size of the different groups analyzed; Tox+, confirmation of the presence of toxic substances in the corpse.

A total of 355 suicide autopsies were retrospectively analyzed from January 2013 to December 2018 at the Institute of Legal Medicine and Forensic Sciences (ILMFC) of the Region of Murcia (Spain). All the autopsies reported in this study included suicide as a cause of violent death according to the WHO International Classification of Diseases (ICD-11) [34]. Autopsies with indeterminate certification of death were not included in this study. In 56.9% of the cases analyzed (n = 202), toxicological findings were found in the corpse (Figure 1). Sociodemographic and clinical characteristics, main suicide mechanisms, the presence and concentration of 26 different toxics, and their analytical techniques were studied.

Suicides were classified according to the method or tool used to consummate the suicidal act. For their study, the different mechanisms of suicide were classified into nine groups: Stab wounds, CO poisoning, gunshot wounds, hanging, drug overdose, jumping from a height, drowning, suffocation, traffic accident, or standing in front of a moving vehicle. The study was conducted in accordance with the Declaration of Helsinki, and the Ethics Committee approved the protocol of the University of Murcia (ID 2606/2019).

### 2.2. Chemical–Toxicological Analytical Techniques

**Figure 2:**
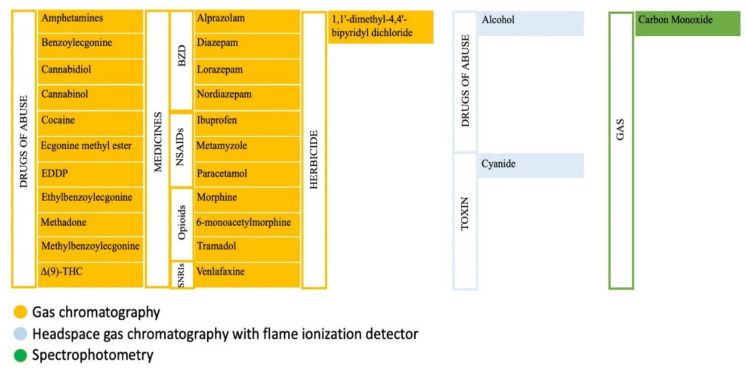
Classification of the different toxics analyzed in this study and the analytical techniques used for their laboratory determination. EDDP, 2-etileno-1,5 dimetil 3-3 difenilpirrolodina; Δ(9)-THC, Δ(9)-tetrahidrocannbinol.

The analytical determination of 26 substances in the corpse was only detected in the femoral and peripheral venous. All the analytical techniques used to detect substances in blood were performed after the solid extraction of the sample using bond elut™ column extraction (Agilent Technologies). Headspace gas chromatography and gas chromatography detected abused drugs and medicine [35,36,37,38,39,40,41,42] as alcohol and cyanide [43,44]. Carbon monoxide (CO) was detected using spectrophotometry [45] (Figure 2).

In cases subject to judicial instruction or fiscal information proceedings, the toxics were examined at the National Institute of Toxicology and Forensic Science (Spain).

A single intoxication was considered when toxics and their metabolites were found in the corpse. This single intoxication occurs in the case of cocaine and its metabolites such as benzoylecgonine, ecgonine methyl ester, ethylbenzoylecgonine, and methylbenzoylecgonine; methadone (metabolite: EDDP); and morphine and with heroin (metabolite: 6-monoacetylmorphine). Polyconsumption was considered when two or more different toxics were found in the corpse.

The concentrations were expressed in milligrams per liter, except for alcohol concentrations (grams per liter) and carbon monoxide concentrations (percentage of carboxyhemoglobin in the blood). The concentrations of the different toxics were analyzed and compared with lethal dose values of postmortem blood [46,47].

### 2.3. Statistical Analysis

Demographic data and results were obtained and analyzed using SPSS 24.5 (SPSS software Inc., Chicago, IL) in a database (Microsoft Access 2.0; Microsoft Corporation, Seattle, WA). All results were expressed as the mean or percentage ± standard deviation (SD). Pearson’s X^2^ and the similar results of the two-tailed Fisher test were used to compare classified variables between groups, and Student’s T-Test was used to compare means of independent samples and establish the level of significance at 95%. *p*-values below 0.05 were considered significant.

## 3. Results

### 3.1. Suicides in the Study Period Analyzed

**Figure 3:**
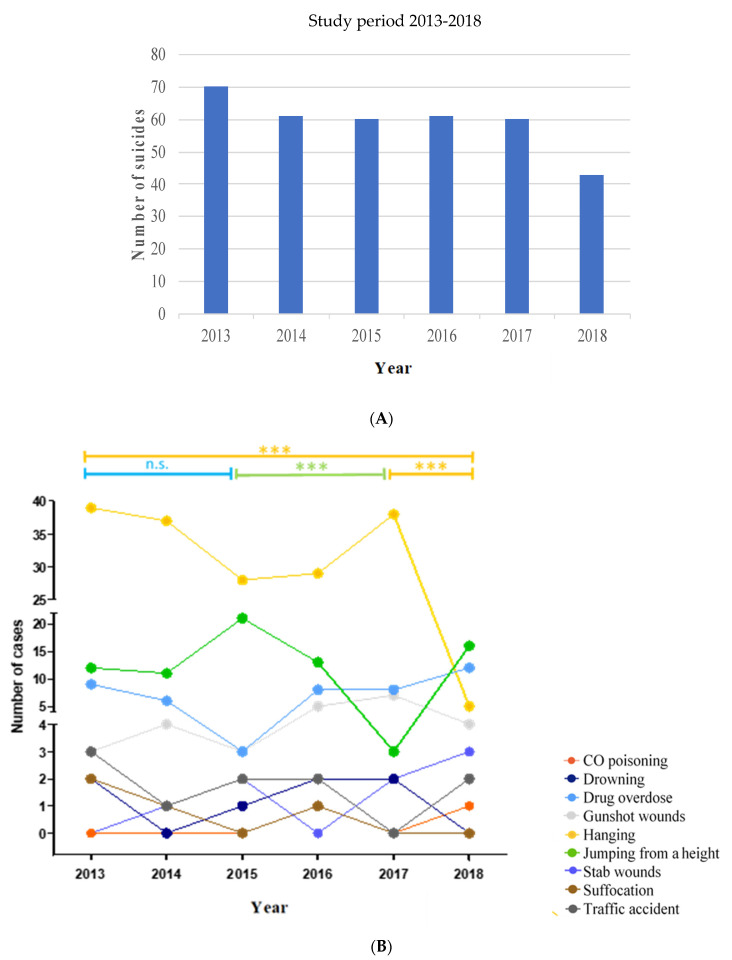
Analysis of the suicides analyzed in the time period of this study. (**A**) Number of suicides that occurred in the analyzed study period. (**B**) Classification of the number of suicides and the suicide mechanism analyzed. n.s: not statistically significant. *** *p* < 0.001.

The number of suicides was classified by years and the suicide mechanism used (Figure 3). To compare the total number of suicides at the beginning and the end of the period analyzed (2013 and 2018), a decrease in cases (38.5%) was observed (Figure 3A). A decrease of 12.9% in cases of suicides was observed when comparing 2014 with 2013 and 30% when comparing 2018 and 2017 (30%).

The most represented suicide mechanisms were hanging, jumping from a height, and drug overdose (Figure 3B). Hanging was the most represented mechanism of suicide in our autopsy case study despite a statistically significant reduction when comparing the numbers of 2013 and 2018 (39 and 5 cases; *p* < 0.001; OR = 9.310; 95% CI: 0.270 to 26.503). Similarly, a statistically significant reduction was observed when comparing the cases registered in 2017 with 2018 (*p* < 0.001; OR = 12,782; 95% CI: 4.378 to 37.315).

The second most represented was jumping from a height, reaching a maximum of 21 cases in 2015 and exhibiting a decrease in cases until 2017 (*p* < 0.001; OR = 95; 95% CI: 2.644 to 34.136). Drug overdose was the third mechanism represented with a maximum of 12 cases in 2018, not exhibiting any statistically significant trend.

Other mechanisms of suicide observed in our autopsy cases were CO poisoning, drowning, gunshot, stab wounds, suffocation, and traffic accident, and there was no statistically significant difference in any case in the period analyzed. Finally, the relationship between the number of cases per year and the sex of the suicide victims was analyzed, and statistically significant differences were not found (X^2^ = 0.195).

### 3.2. Analysis of Suicide Mechanism, Toxicological Findings, and Sex

The relationship between suicide mechanism, toxicological findings, and sex was analyzed (Table 1). A statistically significant relationship between gender and the suicide mechanism was observed (X^2^ = 33.777; *p* < 0.010).

Of the suicide cases, 273 suicides corresponded to males (76.9%) and 82 cases to females (23.10%). The mean age of the total autopsy cases was 52.0 ± 17.4 (mean years ± SD), and the age range was between 12 and 93 years. Hanging (49.6%) was the most represented suicide mechanism, followed by jumping from a height (21.4%), drug overdose (13%), gunshot wounds (7.3%), traffic accident (2.8%), stab wounds (2.25%), drowning (2%), suffocation (1.1%), and CO poisoning (0.6%). The analysis by sex showed that men are more represented than women. In most suicide mechanisms (drowning, drug overdose, gunshot, and traffic accident), men have a higher average age than women, except for suicide by suffocation, where women had a higher average age than men. However, no statistically significant differences were observed in any of the cases.

Suicides with positive toxicology were observed in 202 victims (56.9%), 155 (76.7%) men, and 47 (23.2%) women. The most represented suicide mechanisms in suicides with positive toxicology were hanging (55%), drug overdose (22.7%), and jumping from a height (17.8%). In men, the most represented suicide mechanisms were hanging (50.3%), while women were represented in 29.7%), this difference being statistically significant (*p* = 0.019; OR = 2.388; 95% CI:1.186 to 4.808). On the contrary, women were more represented in the jumping from a height group than men (29.7% vs. 14.2%; *p* = 0.028; OR = 0.390; 95% CI:0.180 to 0.843). In all cases, the mean ages were similar in both groups, with slightly lower ages being observed in traffic accidents (45.17 ± 18.78: mean ± SD) and jumping from a height (48.36 ± 15.04: mean ± SD). In drug overdose suicides, the difference between the mean age of men (53.78 ± 15.13) and women (45.00 ± 17.33) is marginally significant (*p* = 0.090).

In suicides with negative toxicology, the most frequent suicide mechanisms were hanging (54.5%) and jumping from a height (26.1%). Statistically positive differences between men and women were found in these two suicide mechanisms. Men were more represented than women in hanging (61%; *p* = 0.007), while for jumping from a height, women were more represented (54.2%; *p* < 0.001).

### 3.3. Analysis of Suicide Mechanisms and the Number of Toxics Found

For the analysis of suicides with positive toxicology (n = 202), two groups were generated based on the number of toxics found (1 toxic and ≥2 toxics) and the suicide mechanism used (Table 2). A total of 65.8% of positive toxicology corpses showed the presence of a single toxic, and in 4.2%, two or more toxics were found.

The presence of a single toxic was found more frequently in cases of hanging (53.4%), jumping from a height (21.8%), and drug overdose (12.8%), while the presence of two or more toxics was more frequent in suicides by drug overdose (42%), hanging (30.4%), and jumping from a height (10.1%). Then, the drug overdose cases were analyzed, and the presence of ≥2 toxic substances in the corpse was observed more frequently in a statistically significant manner than the presence of one toxic (42% vs. 12.8%; *p* < 0.001). On the contrary, in the mechanisms of suicide by hanging and jumping from a height, the presence of a single toxic was statistically more frequent than more than one toxic (53.4% vs. 30.4%; *p* = 0.003 and 21.8% vs. 10.1%: *p* = 0.052, respectively).

**Figure 4:**
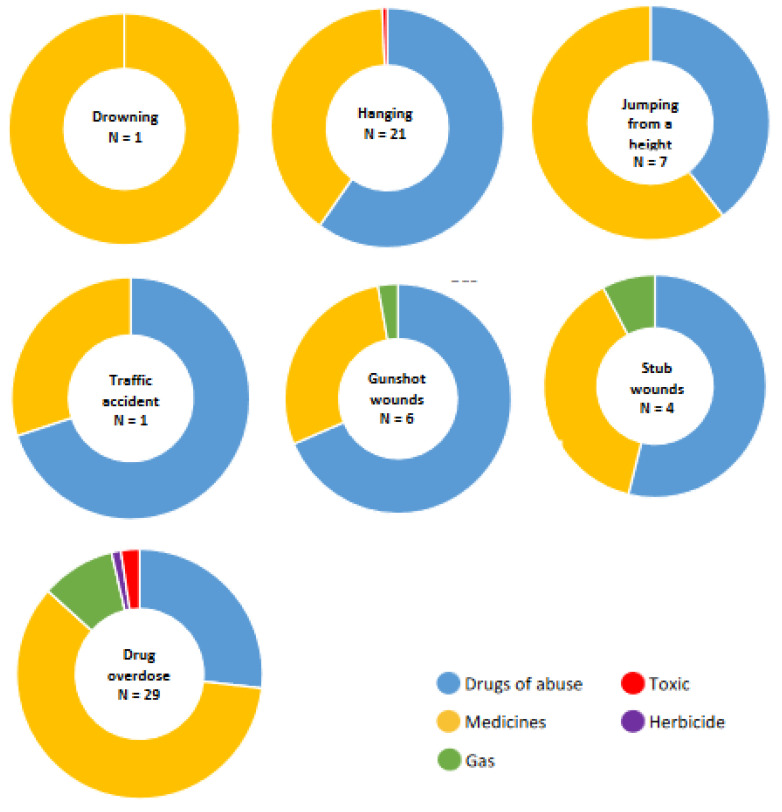
Suicide mechanisms.

Subsequently, an analysis of the types of toxics found in the different mechanisms of suicide was carried out (Figure 4). Drugs of abuse and medicines were the main causes in all suicide mechanisms analyzed with two or more toxics. Intoxication by gas was present in gunshot, stab wounds, and drug overdose cases. Finally, in drug overdose cases, suicides were observed with the toxic cyanide and the herbicide 1-1′-dimethyl-4,4′-bipyridyl dichloride.

### 3.4. Analysis of the Types of Toxics Found According to the Sex of the Suicide Victims

As shown in Table 3, drugs of abuse were the most frequent types of toxics found (48.7%), being statistically more significantly represented in men than women (55.5% and 24.3%, respectively; *p* < 0.001; OR = 3.882; 95% CI: 2.165 to 6.962). Alcohol was the most represented drug of abuse (21.7%), with a slightly higher frequency in men than in women, with this difference bordering on statistical significance (23.9% vs. 13.5%; *p* = 0.057). Subsequently, cocaine and its metabolites were present in 16.3%, with statistically significant differences between men and women (20.2% vs. 2.7%; *p* < 0.001; OR = 9.636; 95% CI: 2.295 to 40.462). Cannabinoids (8.3%) and methadone and their metabolites (1.8%) were also present in the analyzed autopsy cases, but no differences in presence were observed according to sex.

The medicines were found in 158 (46.9%) cases, being statistically more significantly represented in women than men (70.3% and 40.3%, respectively; *p* < 0.001; OR = 0.232; 95% CI: 0.143 to 0.377). Benzodiazepines were the most represented drug group (30.9%), statistically more significantly represented in women than men (43.2% and 27.4%, respectively; *p* = 0.011; OR = 0.495; 95% CI: 0.290 to 0.844). It should be noted that the most frequent type of benzodiazepine was nordiazepam (14.8%), followed by lorazepam (8.0%), alprazolam (5.0%), and finally diazepam (3.0%). They were equally present in men and women in most cases, except for lorazepam, which was more represented in women than in men (14.9% vs. 6.1%; *p* = 0.026; OR = 0.371; 95% CI: 0.164 to 0.839).

The analysis of nonsteroidal anti-inflammatory drugs (NSAIDs) represented 3.9% of all medicines. Paracetamol (2.7%) was the most represented, followed by ibuprofen (0.9%). No statistically significant differences were observed between the sexes.

Serotonin and norepinephrine reuptake inhibitors (SNRIs) such as venlafaxine were present in 9.2%. The presence of opioids was more frequent in women (6.8%) than in men (1.9%), and this difference was statistically significant (*p* = 0.045; OR = 0.267; 95% CI: 0.075 to 0.950). Finally, carbon monoxide (3.3%), cyanide (0.9%), and 1,1′-dimethyl-4-4′-bipyridyl dichloride (0.3%) were detected.

### 3.5. Toxic Combinations Found in Suicides

Subsequently, a study was conducted to determine the most common combinations of toxics in our suicide cases (Table 4). Polyconsumption was detected in 63 suicide cases, involving two to six different substances. It should be noted that alcohol was the most consumed toxic in combination with other toxics cases (50.79%), followed by nordiazepam, cocaine, and venlafaxine. Cocaine was found along with benzodiazepines, methadone, morphine, and NSAIDs. Other combinations were also present, such as cocaine with alprazolam and cannabis as well as nordiazepam with diazepam. Venlafaxine was detected with the benzodiazepines alprazolam or lorazepam, or cannabis.

### 3.6. Types of Toxics Found in the Different Mechanisms of Suicide

**Figure 5:**
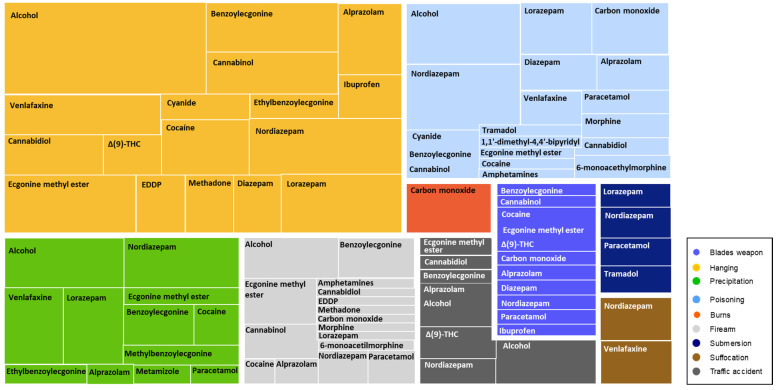
Representative scheme of the different toxics found in the autopsy according to the suicide mechanism. The size of the box for each toxic represents its frequency of presence in the autopsies.

Finally, the types of toxics found in the different mechanisms of suicide were analyzed (Figure 5, Table S1). Benzodiazepines were found with high frequency in suicides due to drug overdose, hanging, and jumping from a height, highlighting that they were more present in men than in women. On the other hand, venlafaxine had a strong presence in suicides by hanging, jumping from a height, and drug overdose.

In all suicide mechanisms, alcohol was present, except for death by submersion and suffocation, where medicines were found. Note that alcohol was detected in 40 hangings, most in men (n = 38). On the other hand, cocaine and its metabolites were highly represented in suicides by hanging and gunshot wounds.

### 3.7. Analysis of Concentrations of the Different Toxics in Drug Overdose Suicides

The toxic concentrations found postmortem in drug overdose suicides were analyzed and compared with lethal reference concentrations (Table 5). In 46 cases classified as drug overdose, toxic substances were found in lethal doses in only 28 cases. Among the toxins most frequently found in lethal toxic doses, lorazepam and carbon monoxide stand out, with seven cases each. Among the benzodiazepines, alprazolam also stands out with three autopsies with lethal doses. Otherwise, morphine was found in lethal doses in three cases, while cyanide was only found in two autopsies, both in lethal concentrations. In the case of drugs of abuse, alcohol, the average concentration of which exceeded 1.2 mg/L, is not in the lethal reference range in any case. Benzoylecgonine was only found in two cases, both in a lethal dose, as is the case with cocaine, which was only found in an autopsy and was also in the lethal range.

## 4. Discussion

Our study aimed to analyze the types, combinations, and concentrations of toxics found in fatal suicide victims with different suicide mechanisms to increase knowledge and establish policies and protocols for detecting, preventing, and avoiding suicide behavior.

According to the World Health Organization, 20 persons attempt suicide for every suicide death, a significant public health problem [7]. Depending on the lethality of regularly used suicide techniques, this ratio varies from country to country [48]. As a result, it has received increased attention in research and public awareness efforts during the last decade and is a strong focus of the international public health community [7]. Most countries have implemented suicide prevention strategies with varied effectiveness [49,50,51]. Today, we can build a complete picture of suicidal behavior due to advances in understanding the factors influencing suicide risk [52,53,54,55].

According to the data obtained from the National Institute of Statistics (INE) in Spain, the number of suicides is higher in men than in women. These data correlate with those of the present study and those carried out in recent years in Spain [38] and Europe [21,56,57,58].

On the other hand, the WHO does not specify or classify suicide methods, and few studies analyze the different mechanisms of suicide by age group and sex [59,60]. This fact makes it difficult to establish the frequency with which suicide methods occur in different populations. Hanging has been the main method used by young men in Europe and Australasia [61,62]. Our results showed that the main method of suicide for men was hanging, verified by other results from Spanish [63,64,65] and European [66,67] regions.

Many suicides presented positive toxicology to one or more toxics in our study. Alcohol was the most frequently found toxicant in all suicide mechanisms except in the mechanisms of burns, submersion, suffocation, and traffic accidents, perhaps because these mechanisms were the ones with the smallest sample size. Alcohol was found in average concentrations above 1.2 mg/L, the toxic range in which decision-making capacity is altered [68,69].

This study observed that intoxication as a mechanism and/or cause of suicide is less present than violent agents in other developed countries [70]. This may be due to the various hospital measures carried out in the minutes or hours close to ingesting the toxic substance (use of antidotes, neutralization, or elimination) that mitigate the damage in the body, avoiding death. This may explain the notably lower percentage of suicides due to intoxication than suicides due to hanging and jumping from a height since, in this study, suicides were analyzed and not suicide attempts.

On the other hand, a bidirectional model between suicidality and drug use has been described since the latter progressively increases the appearance of suicidal ideas [71]. Classically, a dichotomous character has been described in overdose: accidental or suicidal, but there is great difficulty in establishing the intention of this conduct [72]. After drug use, a disinhibiting effect can be generated in the subject, facilitating the transition to the act and the consequent execution of suicidal behaviors [73]. For the latter, it is important to consider the effect of toxic substances in all suicides and not only in suicides due to intoxication, since these hinder the capacity and autonomy of the subject in making decisions [71,74,75,76].

Age did not play any significant role when combined with the population with positive toxicology findings (*p* > 0.05). However, other studies observed an increased suicide rate in older versus younger adolescents due to a higher prevalence of psychopathology, i.e., substance abuse, and higher suicidal intent in the older population. The increased rate in men is less easily explained but may be due to method choice and the higher prevalence and risk associated with conduct disorder in men [77,78]. Our study shows that toxicological findings are present in more than 50% of all suicides, which indicates their significant implication. Among the suicides in which the cause of death is not intoxication, suicides by hanging are the ones that present a higher frequency of toxics, with a more significant number of autopsies with the presence of toxics.

On the other hand, in our autopsy cases, alcohol consumption in autopsies differed in gender (*p* = 0.031), with men who consumed alcohol being higher than women. On the contrary, gender and presence of benzodiazepines were also statistically significant (*p* = 0.018); in this case, the percentage of women who consume benzodiazepines is greater than that of men. The large percentage of deliberate benzodiazepine poisonings with mental health issue diagnoses, particularly among young females, emphasizes the significance of emergency department mental health and suicide risk assessments with proper follow-up referrals [79].

Various studies show that polydrug use is associated with an increased risk of completed suicide [73,75,80,81,82,83]. It should be noted that the majority of deaths in which toxic substances have been found involve the use of multiple toxics, particularly psychotropic drugs in combination with other central nervous system depressants, such as alcohol or psychotropic drugs such as benzodiazepines or venlafaxine. Some studies show that depression combined with alcohol can increase the risk of suicide; psychotropic drugs, both antidepressants and benzodiazepines, are frequently used to treat anxiety and depression [84,85,86,87].

Many suicides in which toxic substances were recorded were related to psychotropic drugs, where one or more benzodiazepines were found, which indicates that a high percentage of the deceased were under treatment. Although the benzodiazepine prospects state that the treatment should be short and not longer than three months, these drugs are prescribed for long periods, even years, without any determining need [88]. In our autopsy cases, serotonin and norepinephrine reuptake inhibitors (SNRIs) were found, specifically venlafaxine, which indicates that the subjects were under treatment with antidepressants. These data could be due to inadequate patient supervision and would confirm the high level of abuse of these substances, often associated with other toxic substances.

In our study, alcohol was found in a high percentage with concentrations exceeding 1.5 mg/L. Alcohol has an essential effect on decision-making and directly correlates with suicidal instinct [68,69]. A study observed in hospitalized patients with alcoholism and depression that 40% had attempted suicide in the previous week, and 70% had attempted it at some point in their lives [89].

Cocaine and/or one of its metabolites were found in our study, accounting for 6.2% of all suicides. The study carried out by Ortega Pérez et al. [90] shows that cocaine has a blood half-life of between 40 and 90 min, so it disappears from the blood within a few hours. However, the determination in femoral blood indicated that the cocaine was consumed in the minutes close to death. Cocaine is transformed into several different metabolites, of which benzoylecgonine is the most common. The elimination rate of this toxic depends on the individual’s cholinesterase concentration and activity. Benzoylecgonine has a 6–8 h plasma half-life and its ecgonine methyl ester 3–8 h. Both metabolites have a longer plasma half-life than cocaine, which facilitates their detection and makes them two metabolites of great forensic interest [91].

## 5. Conclusions

In summary, our study shows how the number of suicides in men is higher than in women. In addition, more than 50% of the suicides analyzed present positive toxicology, which highlights the vital role of toxic substances in suicidal decision-making. It should also be noted that the percentage of autopsies of women in which toxic substances were found is higher (57.31%) than the percentage of men (52.57%). The percentage of positive toxicological findings is higher in men for the following suicide mechanisms: hanging, firearm, burns, and traffic accidents. In contrast, the percentages are higher in women for the mechanisms of intoxication, jumping from a height, and suffocation.

Drugs of abuse were the toxins most frequently found in the suicides studied, with alcohol being the most present toxin, being found more often in men (23.5%) than in women (13.51%). Psychopharmaceuticals were the second most found toxic group. Unlike alcohol, the findings of psychotropic drugs were higher in women (43.23%) than in men (27.37%).

The toxicological findings show the polyconsumption of toxins, the most frequent association of toxins being alcohol with other toxins, with benzodiazepines being the that most frequently observed group of drugs. In second place is the simultaneous use of psychoactive drugs, specifically benzodiazepines and venlafaxine (SNRIs). The percentage of benzodiazepines and venlafaxine found in therapeutic doses was greater than the percentage of these drugs found in toxic doses. This fact indicates the high number of subjects undergoing pharmacological treatment and the adequate follow-up these patients should have.

Due to the importance of toxics and drugs in suicide, in the future, it would be interesting to compare the toxicological results obtained in cases of suicide with those of non-suicide since these data would shed light on the influence of the intake of toxics and drugs in suicide. Toxicological analyses are fundamental to understanding consumption patterns and establishing strategies and protocols for detecting and preventing suicide.

## Figures and Tables

**Table 1:** Analysis of the frequency of suicides according to suicide mechanism, toxicological findings, and sex.

Suicide Mechanisms	Total Suicide Cases				Positive Toxicology				Negative Toxicology			
	Total *N* = 355, n (%) (Mean ± SD) *	Male	Female	P1	Total	Male	Female	P2	Total	Male	Female	P3
**TOTAL**	355 (100) 52.09 ± 17.47	273 (76.9) 53.02 ± 17.85	82 (23.1) 49.00 ± 15.84	0.067	202 (56.9) 50.60 ± 15.33	155 (76.7) 50.83 ± 15.13	47 (23.2) 49.85 ± 16.13	<0.001 ^a^ 0.702	153 (43.1) 54.07 ± 19.82	118 (77.1) 55.91 ± 20.611	35 (22.8) 47.86 ± 15.60	0.034 ^d^
**CO poisoning**	2 (0.6) 57.00 ± 2.82	2 (100) 57.00 ± 2.82	- -	- -	1 (0.5) 55.00 ± 0.00	1 (0.6) 59.78 ± 12.82	- -	- -	1 (0.6) 59.00 ± 0.00	1 (0.8) 59.00 ± 0.00	- -	- -
**Drowning**	7 (2.0) 66.43 ± 16.64	5 (71.4) 72.20 ± 16.42	2 (28.5) 52.00 ± 0.00	- 0.161	1 (0.5) 52.00 ± 0.00	- -	1 (2.1) 52.00 ±0.00	- -	6 (3.9) 68.83 ± 16.84	5 (4.2) 72.20 ±16.42	1 (2.8) 52.00 ± 0.00	1.000
**Drug overdose**	46 (13.0) 51.11 ± 16.16	32 (69.6) 53.78 ± 15.13	14 (30.4) 45.00 ± 17.33	0.090	46 (22.7) 51.11 ± 16.16	32 (20.6) 53.78 ± 15.13	14 (29.0) 45.00 ± 17.33	0.233 0.090	- -	- -	- -	- -
**Gunshot wounds**	26 (7.3) 55.15 ± 17.65	25 (96.2) 55.64 ± 17.84	1 (3.8) 43.00 ± 0.01	- -	12 (5.9) 51.58 ± 18.84	12 (7.7) 51.58 ±18.84	- -	- -	14 (9.1) 58.21 ± 16.65	13 (11.0) 59.38 ± 16.72	1 (2.8) 43.00 ±0.00	0.192
**Hanging**	176 (49.6) 53.11 ± 17.60	150 (85.2) 53.30 ± 17.52	26 (14.7) 52.04 ± 18.35	0.737	92 (45.5) 51.15 ±14.95	78 (50.3) 51.01 ± 14.47	14 (29.7) 51.93 ± 18.00	0.019 ^b^ 0.834	84 (54.9) 55.26 ± 19.98	72 (61.0) 55.78 ± 20.10	12 (34.2) 52.17 ± 19.55	0.007 ^d^ 0.564
**Jumping from a height**	76 (21.4) 47.39 ± 17.69	43 (56.6) 46.88 ± 20.18	33 (43.4) 48.06 ± 14.09	0.775	36 (17.8) 48.36 ± 15.04	22 (14.2) 45.91 ±15.81	14 (29.7) 52.21± 3.40	0.028 ^c^ 0.152	40 (26.1) 46.53 ± 19.93	21 (17.8) 47.90 ± 24.30	19 (54.2) 45.00 ±14.14	<0.001 ^e^ 0.651
**Stab wounds**	8 (2.2) 56.38 ± 15.96	6 (75.0) 57.83 ± 18.57	2 (25.0) 52.00 ± 2.82	0.688	6 (2.9) 51.33 ±10.59	5 (3.2) 51.60 ±11.82	1 (2.1) 50.00 ± 0.00	- -	2 (1.3) 71.50 ± 24.74	1 (0.8) 89.00 ±0.00	1 (2.8) 54.00 ± 0.00	0.406-
**Suffocation**	4 (1.10) 54.25 ± 15.69	2 (50.0) 49.50 ± 0.7	2 (50.0) 59.00 ± 25.45	0.650	2 (0.9) 59.00 ± 25.45	- -	2 (4.2) 59.00 ± 25.45	- -	2 (1.3) 49.50 ± 0.70	2 (1.6) 49.50 ± 0.70	- -	- -
**Traffic accident**	10 (2.8) 51.10 ± 17.51	8 (80.0) 53.88 ± 18.52	2 (20.0) 40.00 ± 7.07	0.345	6 (2.9) 45.17 ±17.52	5 (3.2) 47.20 ± 18.78	1 (2.1) 35.00 ± 0.00	1.000-	4 (2.6) 60.00 ± 15.25	3 (2.5) 65.00 ±14.10	1 (2.8) 45.00 ± 0.00	1.000 -

Subgroup size. SD, standard deviation. CO, carbon dioxide. (-), no case analyzed. * The main age of all groups analyzed is expressed as year mean ± SD. P1-3, comparisons were made between sexes with different sample sizes and mean ages. *p* < 0.05 was considered statistically significant and is marked in bold. ^a^ *p* < 0.001; OR = 0.400; 95% CI: 0.272 to 0.590; ^b^ *p* = 0.019; OR = 2.388; 95% CI: 1.186 to 4.808; ^c^ *p* = 0.028; OR = 0.390; 95% CI: 0.180 to 10.843; ^d^ *p* = 0.007; OR = 3.000; 95% CI: 1.362 to 6.610; ^e^ *p* < 0.001; OR = 0.182; 95% CI: 0.081 to 0.412.

**Table 2:** The number of toxics found in the corpse with different suicide mechanisms.

	Positive Toxicology, *n* = 202		
Suicide mechanisms	1 toxic *n* = 133, (65.8%)	≥2 toxics *n* = 69, (34.2%)	*p*
**CO poisoning (n = 1)**	1 (0.7)	0 (0.0)	-
**Drowning (n = 1)**	0 (0.0)	1 (1.5)	0.342
**Drug overdose (n = 46)**	17 (12.8)	29 (42.0)	**<0.001 ^a^**
**Gunshot wounds (n = 12)**	6 (4.51)	6 (8.70)	0.346
**Hanging (n = 92)**	71 (53.4)	21 (30.4)	**0.003 ^b^**
**Jumping from a height (n = 36)**	29 (21.8)	7 (10.1)	0.052
**Stab wounds (n = 6)**	2 (1.5)	4 (5.8)	0.183
**Suffocation (n = 2)**	2 (1.5)	0 (0)	0.548
**Traffic accident (n = 6)**	5 (3.8)	1 (1.5)	0.666

*n*: total number of suicides in each group. *p* < 0.05 was considered statistically significant and is marked in bold. ^a^ *p* < 0.001; OR = 0.202; 95% CI: 0.101 to 0.406; ^b^ *p* < 0.003; OR = 2.618; 95% CI: 1.414 to 4.845.

**Table 3:** Analysis of the types of toxics found according to the sex of the suicide victims.

Types of Toxics		Total N = 202, (%)	Male n = 273, (%)	Female n = 82 (%)	*p*
**Medicines**	**Total**	158 (46.9)	106 (40.3)	52 (70.3)	<0.001 ^a^
	**Benzodiazepines**	104 (30.9)	72 (27.4)^c^	32 (43.2)	0.011 ^b^
	Alprazolam	17 (5.0)	11 (4.2)	6 (8.1)	0.224
	Diazepam	10 (3.0)	8 (3.0)	2 (2.7)	1.000
	Lorazepam	27 (8.0)	16 (6.1)	11 (14.9)	0.026 ^c^
	Nordiazepam	50 (14.8)	37 (14.1)	13 (17.6)	0.462
	**SNRIs** Venlafaxine	31 (9.2)	20 (7.6)	11 (14.9)	0.068
	**NSAIDs**	13 (3.9)	9 (3.4)	4 (5.4)	0.493
	Ibuprofen	3 (0.9)	2 (0.8)	1 (1.4)	0.526
	Metamizole	1 (0.3)	1 (0.4)	0 (0.0)	-
	Paracetamol	9 (2.7)	6 (2.3)	3 (4.0)	0.418
	**Opioids**	10 (2.9)	5 (1.9)	5 (6.8)	0.045 ^d^
	Morphine	5 (1.5)	3 (1.1)	2 (2.7)	0.303
	6-monoacetylmorphine	3 (0.9)	2 (0.8)	1 (1.4)	0.526
	Tramadol	2 (0.6)	0 (0.0)	2 (2.7)	-
**Drugs of abuse**	**Total**	**164 (48.7)**	**146 (55.5) ^a^**	**18 (24.3)**	**<0.001 ^e^**
	**Alcohol**	73 (21.7)	63 (24.0)^b^	10 (13.5)	0.057
	**Amphetamines**	2 (0.6)	1 (0.4)	1 (1.4)	0.391
	**Cocaine and metabolites**	55 (16.3)	53 (20.2)	2 (2.7)	0.000 ^f^
	Cocaine	13 (3.9)	13 (5.0)	0 (0.0)	-
	Benzoylecgonine	21 (6.2)	20 (7.6)	1 (1.4)	0.056
	Ecgonine methyl ester	18 (5.3)	17 (6.5)	1 (1.4)	0.138
	Ethylbenzoylecgonine	2 (0.6)	2 (0.8)	0 (0.0)	-
	Methylbenzoylecgonine	1 (0.3)	1 (0.4)	0 (0.0)	-
	**Cannabinoids**	28 (8.3)	23 (8.7)	5 (6.8)	0.642
	Δ(9)-THC	2 (0.6)	2 (0.8)	0 (0.0)	-
	Cannabidiol	9 (2.7)	7 (2.7)	2 (2.7)	1.000
	Cannabinol	17 (5.0)	14 (5.3)	3 (4.0)	1.000
	**Methadone and metabolites**	6 (1.8)	6 (2.3)	0 (0.0)	0.343
	Methadone	3 (0.9)	3 (1.1)	0 (0.0)	-
	EDDP	3 (0.9)	3 (1.1)	0 (0.0)	-
Gas	Carbon monoxide	11 (3.3)	8 (3.0)	3 (4.0)	0.712
Toxin	Cyanide	3 (0.9)	2 (0.8)	1 (1.4)	0.526
Herbicide	1,1′-dimethyl-4,4′-bipyridyl dichloride	1 (0.3)	1 (0.4)	0 (0.0)	-

*N*, total number of suicides; NSAIDs, nonsteroidal anti-inflammatory drugs; EDDP, 2-ethylidene-1,5-dimethyl-3,3-diphenylpyrrolidine; SNRIs, serotonin and norepinephrine reuptake inhibitors; Δ(9)-THC, Δ9- tetrahydrocannabinol. Comparisons were made between male and female. ^a^*p* < 0.001; OR = 0.232; 95% CI: 0.143 to 0.377; ^b^ *p* = 0.011; OR = 0.495; 95% CI: 0.290 to 0.844; ^c^ *p* = 0.026; OR = 0.371; 95% CI: 0.164 to 0.839; ^d^ *p* = 0.045; OR = 0.267; 95% CI: 0.075 to 0.950; ^e^ *p* < 0.001; OR = 3.882; 95% CI: 2.165 to 6.962; ^f^ *p* < 0.001; OR = 9.636; 95% CI: 2.295 to 40.462.

**Table 4:** Frequency of the combinations of toxics found in the suicides.

Combinations of Toxics, n (%)					Suicides, *n* = 63
Alcohol+	Alprazolam				1
		+Cocaine			1
		+Nordiazepam			1
		+Nordiazepam + Diazepam + Cannabis			1
	Cannabis				1
	Cocaine				5
		+Cannabis			3
		+Amphetamines + Paracetamol			1
	Diazepam	+Nordiazepam			3
				Paracetamol + Venlafaxine	1
	Carbon monoxide				3
		+Cannabis			1
		+Paracetamol			1
	Lorazepam				3
	Morphine		+Cannabis		1
	Nordiazepam				3
	Paracetamol				1
	Venlafaxine				1
Cannabis+	Nordiazepam				1
Cocaine+	Alprazolam + Cannabis				4
	Nordiazepam	+Lorazepam			1
		+Morphine			1
				+Methadone	1
	Ibuprofen + Paracetamol				1
Cyanide+	Lorazepam				1
Methadone+	Cannabis				1
Carbon monoxide +	Alprazolam				1
	Nordiazepam + Cannabis				1
Morphine+	Amphetamines + Cannabis				1
Nordiazepam+	Diazepam				4
		+Paracetamol + Tramadol			1
	Lorazepam				2
		+Paracetamol + Tramadol			1
Venlafaxine+	Alprazolam				1
	Cannabis				1
	Lorazepam				2
	Nordiazepam				4
		+ Lorazepam			1

BZD: benzodiazepines; n: subgroup size. Suicides from burns and suffocation were not included because no autopsy in this category had more than one toxic.

**Table 5:** Analysis of concentrations of the different toxics in drug overdose suicides.

Types of Toxics		Concentration Postmortem (Mean ± SD)	Concentration Range (Min-Max)	Lethal Reference Dose *	Number of Cases with Lethal Doses (n = 28)
Medicines, n	Benzodiazepines, n				
	Alprazolam, 6	0.270 ± 0.333	0.04–1.00	0.13–2.1	3
	Diazepam, 7	2.433 ± 2.087	0.31–6.85	5–30	1
	Lorazepam, 8	0.531 ± 0.283	0.03–1.00	0.04–0.8	7
	Nordiazepam, 11	1.885 ± 1.289	0.19–4.48	5–30	-
	SNRIs, n				
	Venlafaxine, 6	1.260 ± 1.368	0.05–4.12	1.3–20	2
	NSAIDs, n				
	Paracetamol, 4	15.150 ± 20.345	0.85–50.15	81–1050	-
	Opioids, n				
	Morphine, 4	0.468 ± 0.333	0.10–1.00	0.2–7.2	3
	6-monoacetylmorphine, 2	0.380 ± 0.080	0.30–0.46	-	-
	Tramadol, 1	1.250 ± 0.000	-	1.3–20	-
Drugs of abuse, n	Alcohol, 11	1.269 ± 0.698	0.19–2.32	>3.2	-
	Amphetamines, 1	<0.01		0.5–41	-
	Cocaine and metabolites, n				
	Cocaine, 1	1.08 ± 0.000	-	0.1–330	1
	Benzoylecgonine, 2	1.105 ± 0.275	0.83–1.38	0.05–26	2
	Ecgonine methyl ester, 1	0.12 ± 0.000	-	-	-
	Cannabinoids				
	Cannabidiol, 4	0.485 ± 0.425	0.05–1.00	-	-
	Cannabinol, 2	1.000 ± 0.000	1.00–1.00	-	-
Gas, n	Carbon monoxide, 8	53.125 ± 18.864	5.00–70.00	33–72	7
Toxic, n	Cyanide, 2	31.575 ± 6.575	4.15–38.15	1–100	2
Herbicide, n	1,1′-dimethyl-4,4′-bipyridyl dichloride, 1	3.400 ± 0.000	3.40–3.40	-	-

SD: standard deviation; NSAIDs: nonsteroidal anti-inflammatory drugs; SNRIs: serotonin and norepinephrine reuptake inhibitors; Min: minimum dose; Max: maximum dose. All concentrations are expressed in mg/L, and carbon monoxide is expressed in % carboxyhemoglobin in the blood. * The different reference doses were obtained from previously published data [46].

## Supplementary Materials

The following supporting information can be downloaded at: https://www.mdpi.com/article/10.3390/toxics10060319/s1, Table S1: Analysis of the type of toxics found according to the sex and mechanism of suicide.
