# Supplementary material for: Suicidal Behavior and Its Relationship with Postmortem Forensic Toxicological Findings
Source: Toxics. 2022 Jun 11;10(6):319. doi: 10.3390/toxics10060319 (PMC9229491; doi:10.3390/toxics10060319)
Supplement: Supplementary file 1 [file toxics-10-00319-s001.zip › toxics-1724241-supplementary.pdf]

toxics

Supplementary Materials: Suicidal Behavior and Its Relationship with Postmortem Forensic Toxicological Findings

Aurelia Collados-Ros, Carmen Torres-Sánchez, María D Pérez-Cárceles, Aurelio Luna and Isabel Legaz

Table S1. Analysis of the type of toxics found according to the sex and mechanism of suicide.

		**CO poisoning**			**Drowning**			**Drug overdose**			**Gunshot wounds**			**Hanging**			**Jumping from a height**			**Stab wounds**			**Suffocation**			**Traffic accident**		
		n	**M**	**F**	***n***	**M**	**F**	***n***	**M**	**F**	***n***	**M**	**F**	***n***	**M**	**F**	***n***	**M**	**F**	***n***	**M**	**F**	***n***	**M**	**F**	***n***	**M**	**F**
		1	1	-	4	-	4	82	59	23	35	35	-	143	124	19	50	34	16	10	8	2	2	-	2	10	4	6
**Medicines, *n***	**BZD**																											
	Alprazolam, 17	-	-	-	-	-	-	6	4	2	2	2	-	6	5	1	1	-	1	1	-	1	-	-	-	1	-	**1**
	Diazepam, 10	-	-	-	-	-	-	7	6	1	-	-	-	2	1	1	-	-	-	1	1	-	-	-	-	-	-	-
	Lorazepam, 27	-	-	-	1	-	1	8	4	4	1	1	-	10	7	3	7	4	3	-	-	-	-	-	-	-	-	-
	Nordiazepam, 50	-	-	-	1	-	1	11	9	2	2	2	-	21	15	6	11	8	3	1	1	-	1	-	1	2	2	-
	**SNRIs** Venlafaxine, 31	-	-	-	-	-	-	6	4	2	1	1	-	15	11	4	8	4	4	-	-	-	1	-	1	-	-	-
	**NSAIDs**																											
	Ibuprofen, 3	-	-	-	-	-	-	-	-	-	-	-	-	2	1	1	-	-	-	1	1	-	-	-	-	-	-	-
	Metamizole, 1	-	-	-	-	-	-	-	-	-	-	-	-	-	-	-	1	1	-	-	-	-	-	-	-	-	-	-
	Paracetamol, 9	-	-	-	1	-	1	4	2	2	2	2	-	-	-	-	1	1	-	1	1	-	-	-	-	-	-	-
	**Opioids**																											
	Morphine, 5	-	-	-	-	-	-	4	3	1	1	1	-	-	-	-	-	-	-	-	-	-	-	-	-	-	-	-
	6-monoacetylmorphine, 3	-	-	-	-	-	-	2	1	1	1	1	-	-	-	-	-	-	-	-	-	-	-	-	-	-	-	-
	Tramadol, *n* = 2	-	-	-	1	-	1	1	-	1	-	-	-	-	-	-	-	-	-	-	-	-	-	-	-	-	-	-
**Drugs of abuse, *n***	**Alcohol, 73**	-	-	-	-	-	-	11	9	2	7	7	-	40	38	2	11	6	5	2	1	1	-	-	-	2	2	-
	**Amphetamines, 2**	-	-	-	-	-	-	1	1	-	1	1	-	-	-	-	-	-	-	-	-	-	-	-	-	-	-	-
	**Cocaine and metabolites**																											
	Cocaine, 13	-	-	-	-	-	-	1	1	-	2	2	-	7	7	-	2	2	-	1	1	-	-	-	-	-	-	-
	Benzoylecgonine, 21	-	-	-	-	-	-	2	2	-	4	4	-	10	10	-	3	3	-	1	1	-	-	-	-	1	-	1
	Ecgonine methyl ester, 18	-	-	-	-	-	-	1	1	-	4	4	-	10	10	-	1	1	-	1	1	-	-	-	-	1	-	1
	Ethylbenzoylecgonine, 2	-	-	-	-	-	-	-	-	-	-	-	-	1	1	-	1	1	-	-	-	-	-	-	-	-	-	-
	Methylbenzoylecgonine , 1	-	-	-	-	-	-	-	-	-	-	-	-	-	-	-	1	1	-	-	-	-	-	-	-	-	-	-
	**Cannabinoids**																											
	Δ(9)-THC, 2	-	-	-	-	-	-	-	-	-	-	-	-	2	2	-	-	-	-	-	-	-	-	-	-	-	-	-
	Cannabidiol, 9	-	-	-	-	-	-	4	3	1	1	1	-	3	3	-	-	-	-	-	-	-	-	-	-	1	-	1
	Cannabinol, 17	-	-	-	-	-	-	2	2	-	3	3	-	9	8	1	1	1	-	-	-	-	-	-	-	2	-	2
	**Methadone and metabolites**																											
	Methadone, 3	-	-	-	-	-	-	-	-	-	1	1	-	2	2	-	-	-	-	-	-	-	-	-	-	-	-	-
	EDDP, 3	-	-	-	-	-	-	-	-	-	1	1	-	2	2	-	-	-	-	-	-	-	-	-	-	-	-	-
**Gas, *n***	Carbon monoxide, 11	1	1	-	-	-	-	8	5	3	1	1	-	-	-	-	1	1	-	-	-	-	-	-	-	-	-	-
**Toxic, *n***	Cyanide, 3	-	-	-	-	-	-	2	1	1	-	-	-	1	1	-	-	-	-	-	-	-	-	-	-	-	-	-

**Herbicide, *n***	1,1'-dimethyl-4,4'-bipyridyl dichloride, 1	-	-	-	-	-	-	1	1	-	-	-	-	-	-	-	-	-	-	-	-	-	-	-	-	-	-

M, male, F, female, *N*, total number of toxics. BZDs, benzodiazepines; NSAIDs, nonsteroidal anti-inflammatory drugs; EDDP, 2-ethylidene-1,5-dimethyl-3,3-diphenylpyrrolidine; SNRIs, serotonin and norepinephrine reuptake inhibitors; Δ (9)-THC, Δ9- tetrahydrocannabinol.
